# Introducing Trait Networks to Elucidate the Fluidity of Organismal Evolution Using Palaeontological Data

**DOI:** 10.1093/gbe/evz182

**Published:** 2019-09-05

**Authors:** Etienne Lord, Jananan S Pathmanathan, Eduardo Corel, Vladimir Makarenkov, Philippe Lopez, Frédéric Bouchard, Debashish Bhattacharya, Pierre-Olivier Antoine, Hervé Le Guyader, François-Joseph Lapointe, Eric Bapteste

**Affiliations:** 1 Département d'informatique, Université du Québec à Montréal, Montréal, Québec, Canada; 2 Département de sciences biologiques, Université de Montréal, Montréal, Québec, Canada; 3 Institut de Systématique, Evolution, Biodiversité (ISYEB), Sorbonne Université, CNRS, Museum National d'Histoire Naturelle, EPHE, Université des Antilles, Paris, France; 4 Département de Philosophie, Université de Montreal, Montréal, Quebec, Canada; 5 Department of Biochemistry and Microbiology, Rutgers University, New Brunswick; 6 Institut des Sciences de l'Evolution, cc64, Université de Montpellier, CNRS, Université des Antilles, IRD, EPHE, Montpellier, France

**Keywords:** animal evolution, network, tinkering, complexes, palaeontology, rhinocerotoids

## Abstract

Explaining the evolution of animals requires ecological, developmental, paleontological, and phylogenetic considerations because organismal traits are affected by complex evolutionary processes. Modeling a plurality of processes, operating at distinct time-scales on potentially interdependent traits, can benefit from approaches that are complementary treatments to phylogenetics. Here, we developed an inclusive network approach, implemented in the command line software ComponentGrapher, and analyzed trait co-occurrence of rhinocerotoid mammals. We identified stable, unstable, and pivotal traits, as well as traits contributing to complexes, that may follow to a common developmental regulation, that point to an early implementation of the postcranial Bauplan among rhinocerotoids. Strikingly, most identified traits are highly dissociable, used repeatedly in distinct combinations and in different taxa, which usually do not form clades. Therefore, the genes encoding these traits are likely recruited into novel gene regulation networks during the course of evolution. Our evo-systemic framework, generalizable to other evolved organizations, supports a pluralistic modeling of organismal evolution, including trees and networks.

## Introduction

Organismal evolution is often investigated using phylogenetic approaches, which analyze “characters×taxa” matrices to infer relationships between organismal lineages. The major focus of such, usually tree-based, analyses is generally to determine what groups of organisms derive from a last common ancestor, forming clades, and what are the shared derived features (e.g., the synapomorphies of these clades) are. Thus, phenotypic traits are classically used in morphological phylogenetics, and for retracing the evolution of phenotypic traits along species trees. These trees are increasingly produced from molecular data because trait mapping can reveal the molecular bases of morphological change, as well as illustrate the prevalence of convergent evolution of phenotypic traits (Lee and Palci 2015). Several popular phylogenetic computer programs can also be used to infer ancestral character states, and map their distributions along a reference phylogeny (Ronquist and Huelsenbeck 2003; Csuros 2010; Bouckaert et al. 2014). A critical review of such approaches shows that morphological phylogenetics could further develop by improving models of phenotypic evolution, better taking into account autapomorphies (typically in attempts at tip-dating), and scaling up to accommodate for much broader data sets (Lee and Palci 2015). Whereas these are critical research avenues, our paper is more trivially concerned with developing ways to analyze the distribution of traits across taxa (such as their co-occurrence) using networks, under the assumption that the structure of simple co-occurrence networks can ground original evolutionary interpretations of phenotypes. This is because the recognition and representation of relationships among traits provides a way that might be more directly comparable to corresponding developmental gene networks (and in a way not dependent of known phylogenetic trees). Indeed, while invaluable, in order to describe a broader range of changes and stasis in organisms, phylogenies and character-state mapping can fruitfully be complemented by adopting an even more explicitly system-based perspective (Alon 2006; Wilkins 2007; Yafremava et al. 2013; Esteve-Altava et al. 2015), namely using network approaches that explicitly analyze the interdependency between organismal character states. This view of organisms is deeply rooted in the biological field, as illustrated by the (idealistic) notion of correlation of parts (Cuvier 1812), and its many critical refinements, as it became clear that correlations between animal traits can change in an irregular fashion (Gould 1989). Contra von Baer’s laws of developments, Dollo, De Beer, and others (Gould 1989, 2002; Brigandt 2006) popularized the notion that individual organs can have independent phyletic histories, despite the obvious correlation of parts within any organisms, which represents a clear challenge for the study of organismal evolution. Consistently with this point of view, evo-devo experiments characterized cases of co-options and tinkering of animal traits (Jacob 1977, 2001; Duboule and Wilkins 1998; Carroll 2005; Shubin 2009; Davidson 2010), and showed that structural biases built into genetic and developmental networks (Duboule and Wilkins 1998; Wilkins 2007) can offer relevant explanations of convergences and parallelisms between organisms at the morphological level. These important aspects of organismal evolution challenge traditional analyses (Bolker 2000; Hall 2007; Young and Wagner 2011). Therefore, devising novel approaches to describe and analyze the evolution of relationships between traits constitutes a pivotal question to enhance the understanding of organismal evolution (Wilkins 2007).

Here, we propose to study organismal evolution by using a novel way of enumerating the signal of a given “characters×taxa” matrix. More precisely, these matrices can be recoded into “traits×taxa” matrices to focus on relationships between individual character states. Based on these recoded matrices, “trait networks” can be used to describe and to analyze a rich body of patterns of co-occurrence between the character states that make up the organisms. Thus, trait networks provide a picture of character state combinations, but are not phylogenetic inferences. They are an efficient tool to organize information about various types of co-occurrence of morphological traits in organisms, and to analyze the evolutionary signal associated with these network patterns, while taking advantage of the graph theory methods. This approach differs from the mapping of character states along a reference phylogeny (Tanay et al. 2005) because it does not require the reconstruction of a phylogeny (although it can benefit from the existence of a reference species phylogeny, when one is available). The main focus of our strategy is to detect traits playing important roles in trait networks and to identify groups of traits with remarkable behaviors in order to stimulate hypotheses about the processes affecting the morphology of organisms over the course of evolution. In particular, trait networks can be used to characterize the relative stability of the structural backbone of organisms and to lay out the potential rules of associations for some of their traits. For example, strictly co-occurring morphological traits form “complexes of character states,” which may result from a common developmental regulation or common ancestry. These two interpretations are not mutually exclusive, but the former gains in likelihood, for complexes 1) with traits spatially distributed over the body plans, and 2) distributed across multiple hosts taxa rather than found in a single species. When remarkable (groups of) traits displaying evolutionarily informative patterns in networks do not correspond to synapomorphies of organismal clades, these patterns in trait networks can be used to detect and to highlight evolutionary events and processes that are neither naturally captured nor primarily brought forward in analyses of organismal trees or in character compatibility analyses (Meacham and Estabrook 1985). Yet, trait networks do not aim to replace phylogenetic approaches. Indeed, phylogenetic considerations can further illuminate the outcome of trait networks analyses. For example, traits complexes may be associated with clades, and coincide with synapomorphies of these groups. But trait complexes can also be found in paraphyletic groups of taxa, requiring more complex explanations of their distributions.

## Materials and Methods

### Constitution of the Data Set

We used a matrix derived from (Antoine 2002; Antoine et al. 2003, 2010; Boada-Saña et al. 2008; Becker et al. 2013), including 120 morpho-anatomical characters scored in 15 extinct and six living ceratomorph mammal species (tapirs, rhinoceroses, and their kin), ranging from the last 50 Myr (supplementary data sets S1 and S2, Supplementary Material online, deposited on http://datadryad.org/review?doi=doi:10.5061/dryad.f8886). There was only one missing character state for one taxa in that matrix, otherwise fully documented for a taxonomic sample gathering all suprageneric clades usually recognized within Rhinocerotidae (Becker et al. 2013).

### Construction of the Trait Network

We used the above matrix to construct and analyze the trait network (see main text) with our command line program available at: https://github.com/etiennelord/ComponentGrapher/releases

Unlike us, some users might prefer restricting traits network analyses to traits present in more than one host, since traits found in a single hosts are expected to generate trivial complexes, and would correspond to autapomorphies in phylogenetic analyses, and are usually considered as noninformative in that context.

### Permutation Test

To assess whether the results of the network analyses could have resulted from chance alone, a first permutation test based on the null hypothesis that characters states are randomly distributed among taxa was performed. Namely, this test permutes character states equiprobably in each column of the data matrix in order to break the phylogenetic structure (Archie 1989). Then, a second test based on phylogenetic permutations was carried out to account for phylogenetic autocorrelation between character states, using *k *=* *1.01 as suggested by Lapointe and Garland (2001). New networks were obtained from both of these permuted data sets, from which the corresponding graph statistics were computed. The test values obtained from the actual data matrix were declared significant when the vast majority of the values obtained under the corresponding null hypothesis were more extreme than the original values. For each data set, the number of permutations was set to make sure that the corresponding *P* values could reach a predetermined significance level fixed at 0.05, following a Bonferroni correction for multiple tests.

### Detection of Stable Components

Degree analysis of the network of inclusion (type II) quantifies the relative stability of each trait. Type II in-degree quantifies how many direct neighbors of a given trait point toward this trait, that is, how many traits have a more restricted taxonomic distribution than a focal trait. Type II out-degree quantifies toward how many direct neighbors each individual trait is pointing to, indicating that a focal trait has a more restricted distribution than these neighbors. Very precarious traits have a null in-degree and a positive out-degree. By contrast, stable traits have a higher in-degree and a lower out-degree. To determine which traits are more stable than by chance alone, and are also more stable than expected based on the phylogenetic relationships, another set of permutation tests were performed directly on the network nodes, using the same protocols as described earlier. A trait was considered to be significantly stable when its type II in-degree was more extreme than the vast majority (95%) of in-degrees obtained under the equiprobable and phylogenetic null models.

### Detection of Organismal Fluidity

The extensiveness of trait dissociability was tested by investigating topological features of the type III graph. The density of the graph of type III was computed as follows: $\frac{2k}{N(N-1)}$, where *k* and *N* are the number of edges and traits in the Type III graph, respectively. We also computed the number of triangles and the diameter (longest of the shortest paths between any pair of traits) of the type III graph. The proportion of triangles was computed as the number of triangles divided by the number of possible triplets (three nodes that are linked or not), as follows $\frac{N(N-1)(N-2)}{6}$, with *N* as the number of traits in Type III graphs, in contrast with more classic estimates such as transitivity, which normalizes the count of triangles by dividing it by the number of triads (triplets that are linked). Since our denominator is higher, proportions of triangles are lower than expected with the transitivity formula. The use of the same traits in multiple different morphological combinations, rather than their irremediable replacement in diverging lineages, produces dense type III graphs, with reduced diameters.

## New Approach

We introduce here a new approach and software ComponentGrapher, for the construction and analysis of trait networks. We applied this program to a well-established palaeontological‒neontological data set, focusing on Cenozoic rhinoceroses (Antoine 2002; Antoine et al. 2003). We observed a substantial general dissociability of traits during evolution for these organisms, and identified pivotal and relatively stable traits forming the structural backbone of the rhinocerotoid morphological organization. The general observation that many traits are used repeatedly in distinct combinations in different taxa, which usually do not form a clade provides a novel incentive to further couple developmental and palaeontological studies.

### Introducing Trait Networks

Our method enumerates the signal present in “characters×taxa” matrices to extract patterns of co-occurrence between the character states making the organisms. Thus, it allows one and therefore to generate and to test hypotheses about the evolution of trait relationships during organismal evolution. This method differs from clique/compatibility analysis in its approach, scope, and goals (Salisbury 1999) and produces a picture, instead of an inference, of character state relationships.

**Fig. 1.**
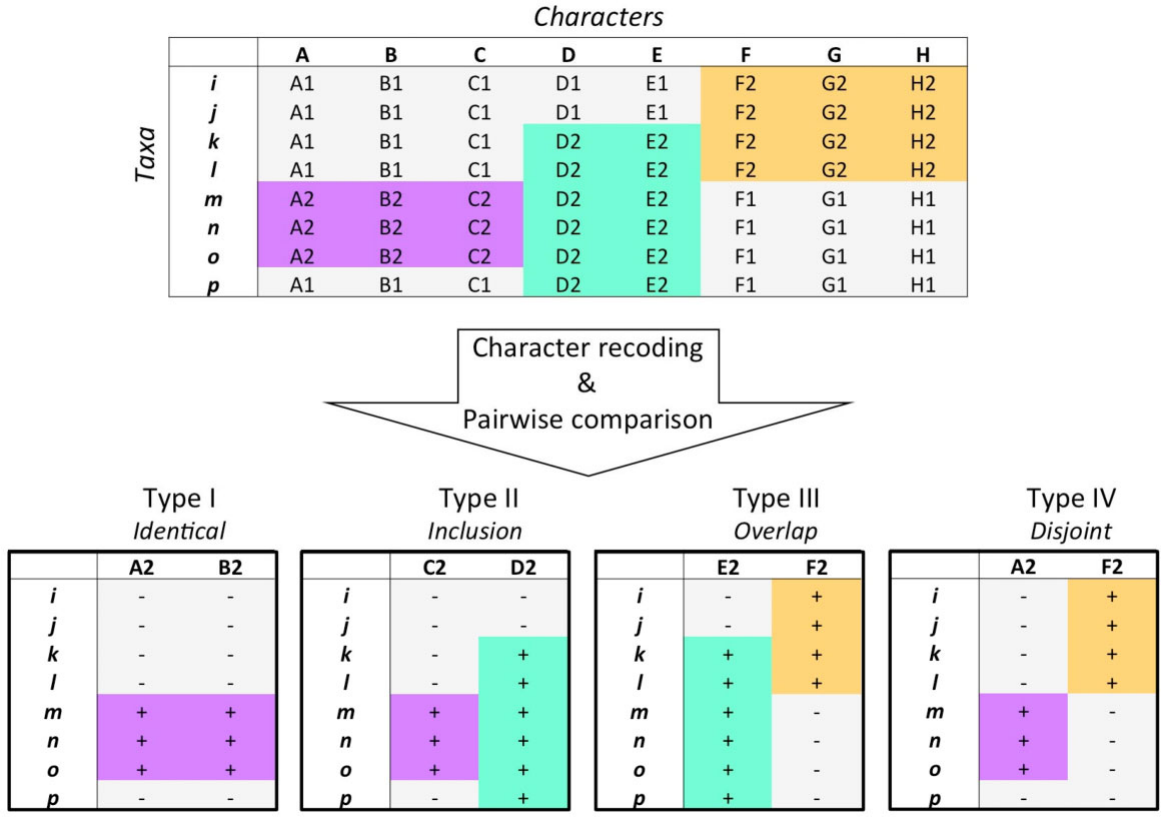
—Principle of the matrix analysis. Our approach exploits existing phylogenetic data matrices featuring taxa as rows and homologous characters as columns. Each original column is replicated in as many new columns as there are character states (e.g., A2, B2), defining a new matrix of taxa by traits, where the presence of each trait is indicated by a “+” and its absence by a “−”. All pairs of columns of this new matrix are then compared with one another, distinguishing four types of distribution of traits across taxa, therefore characterizing four possible types of relationships between all pairs of traits.

**Fig. 2.**
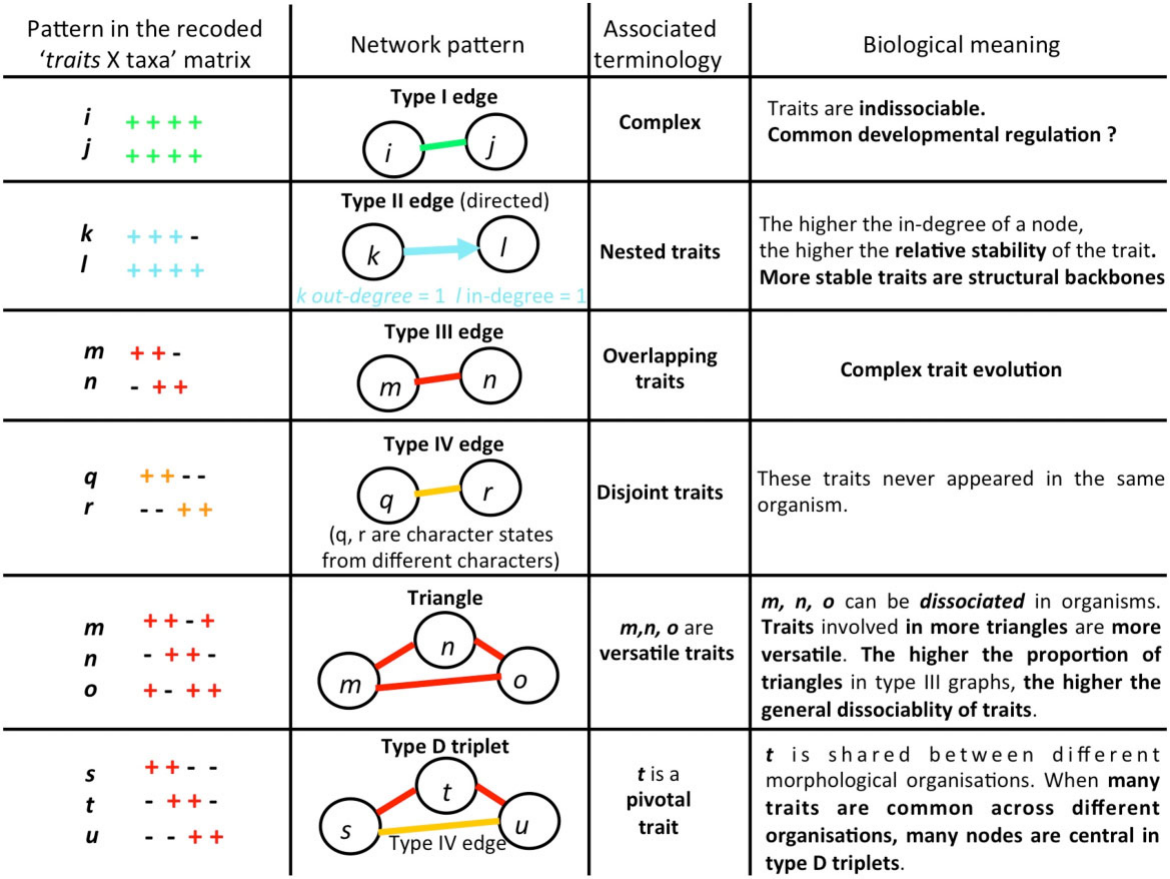
—Some important network patterns and their biological meaning. The first column displays the relationships between a pair of traits (here character states). The second column represents the corresponding network pattern. The third column introduces the terms specifically used to describe and analyze these patterns. The fourth column highlights some possible biological meanings of these patterns.

The main steps of our analyses are described below (see also fig. 1 and supplementary figs. S1 and S2, Supplementary Material online). First, a “characters×taxa” matrix is read by columns. Second, each unique character state associated with a given character is extracted, that is, if an original character had three states (0, 1, 2), this is now split into three character states for which the presence/absence of each of them is scored. Although the effect of recoding multistate characters as binary presence/absence data is problematic for the reconstruction of phylogenetic trees (Maddison 1993; Hawkins et al. 1997; Seitz et al. 2000), this coding is not an issue here because the number of nodes in trait networks is determined only by the number of character states and not by the number of characters. Third, all character states that do not indicate absence are selected. Only those traits (e.g., character states corresponding to a present feature) are considered in subsequent analytical steps. Fourth, the nodes of the trait network are created: each node corresponds to a distinct character state. Fifth, the type of co-occurrence between all pairs of character states from different characters is assessed to build the edges of the trait network (see figs. 1 and 2 and supplementary figs. S1 and S2, Supplementary Material online).

Four different types of relationships were characterized. In a type I relationship, two traits have identical taxon distributions. Because these traits are always found together, they form remarkable sets of features, which we call complexes. Type I edges thus connect two traits with identical host distributions (even if this host distribution is not monophyletic), and define cliques in trait networks. In a type II relationship, one trait shows a broader taxonomic distribution, entirely including that of the other trait. Type II edges are thus directed edges, which connect two traits with nested host distributions. Directed type II edges go from the nested node to the inclusive node, allowing to identify stable traits (characterized by a node with significantly high in-degree for type II edges). When nested host distributions correspond to clades in the species tree, type II edges detect synapomorphies (clade specific groups of traits). When nested host distributions do not correspond to clades, type II edges suggest convergences or independent trait losses/gains, or missing data (it was not the case for the data set studied below). In a type III relationship, two traits have overlapping taxonomic distributions. Thus, type III edges connect traits, which are simultaneously present in some taxa, but have also evolved separately in distinct organisms. Finally, type IV edges connect two traits with mutually exclusive host distributions. Note that with our protocol only pairs of character states associated with distinct characters (not from the same character) are assigned a type IV relationship. Patterns combining type III and type IV edges can automatically detect pivotal traits (i.e., traits that are used in alternative biological organizations in different hosts). Sixth, based on these relationships, the trait network is constructed and stored as a list of nodes and a list of type I–IV edges. The network construction is robust by definition: a given data matrix returns only one network of each type, which is always the same because it represents an exact “picture” of the relationships between character states.

The network is then analyzed to identify the patterns described in figure 2, to compute the two following types of network measures: 1) measures relative to the general topological properties of the trait network, and 2) specific topological properties of each of its nodes. For example, in-degree and out-degree of nodes are computed by counting the number of incoming/outgoing type II edges of each node. Likewise, a node is central in a type D triplet, only if, within such a triplet, this node is connected to two distinct neighbor nodes by a type III edge. Because all network measures used in our analyses rely on exact graph metrics and not on heuristics, the values inferred from the network analyses are also robust. Moreover, permutation tests are used to assess the statistical significance of these network values. First, a null model of uncoordinated evolution is used, whereby all character state evolve independently. Then, a phylogenetic null model, in which the states for each character are correlated is used. These tests comprise a permutation of the states for each character. In the first model, character states are permutated equiprobably across organisms (Archie 1989; Faith and Cranston 1991), whereas in the phylogenetic model, the character states are permuted according to the phylogenetic distances between organisms, as proposed by Lapointe and Garland (2001). Thus, a trait can be considered significantly stable when its type II in-degree is more extreme than the vast majority (95%) of in-degrees obtained under the equiprobable and phylogenetic null permutation models.

### Introducing and Interpreting Some Trait Network Patterns

**Fig. 3.**
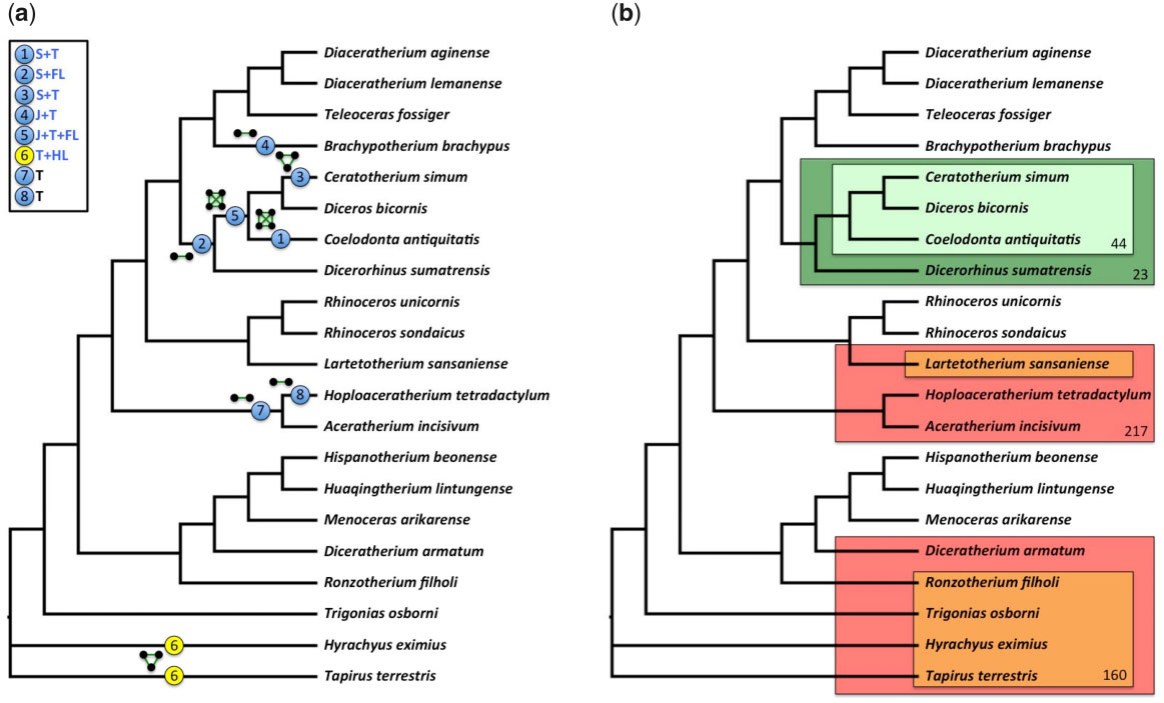
—Composite phylogenetic tree of selected Rhinocerotidae, resulting from the parsimony analyses of (Antoine 2002; Antoine et al. 2003; Boada-Saña et al. 2008; Antoine et al. 2010), based on 282 cranio-mandibular, dental, and postcranial characters, depicting: (*a*) eight trait complexes. Each complex is represented by its corresponding motif (each node represents a trait, each green edge represents the type I relationships between two traits) along the phylogeny, based on its taxonomic distribution. Each complex is also identified by a circled number; blue circles representing complexes shared by a common ancestor and all its descendants (putative synapomorphy), yellow circles representing a complex whose distribution does not map simply onto the phylogeny (homoplasy). The top left squared box identifies the distribution of complexes over the main regions of the rhinocerotoid body plan (S, skull; T, teeth; J, jaw; BP, body plan; FL, forelimb; and HL, hind limb). Blue letters highlight complexes of traits from different regions. (*b*) Phylogeny of Rhinocerotidae showing two exemplary traits with type II relationships. The distribution of trait 44 is nested in that of trait 23 (clade within clade). The distribution of trait 160 is nested in that of trait 217 (nonclade within nonclade). 23: Frontal bone: aspect|‘rugose’; 44: Corpus mandibulae: base|‘very convex’; 160: Lower molars: hypolophid|‘transverse’; 217: Astragalus: orientation trochlea/distal articulation|‘very oblique’.

**Fig. 4.**
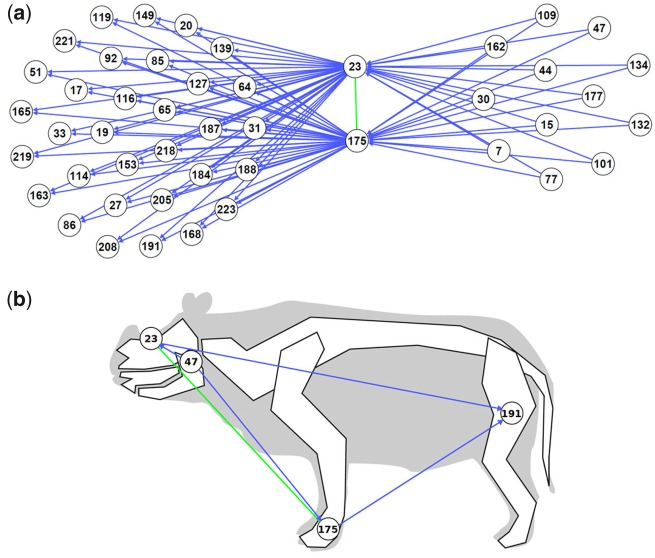
— a) Example of a complex, involved in type II relationships. Nodes 23 and 175 forming the complex are directly connected by a green edge (type I). The distribution of these nodes is nested within the distribution of 33 other nodes (to the left, connected by directed type II edges), and includes the distribution of 12 other nodes (to the left, connected by directed type II edges). b) A selection within these nodes, mapped onto the body plan of rhinos. Node 23: Frontal bone: aspect|‘rugose’; node 175: Pyramidal: distal facet for semilunate|‘L-shaped’; node 47: Ramus|‘inclined backward and upward’; node 191: Femur: trochanter major|‘low’.

Simple motifs with evolutionary significance can be exactly searched for in trait networks. We focused on several of them (fig. 2). Traits connected by type I edges are always associated in organisms. Therefore, such tight associations, particularly when they occur in multiple organisms, compel us to look for explanations, such as common developmental regulations affecting the genes coding for these traits, in particular when these pieces of the morphological toolkit were a priori assumed to evolve independently. For example, the rugose frontal bone (node 23), and the “L-shaped” distal facet for semilunate pyramidal bone (node 175) are always found together, and exclusively so in *Ceratotherium simum* (white rhinoceros, recent), *Diceros bicornis* (black rhinoceros, recent), *Dicerorhinus sumatrensis* (Sumatran rhinoceros, recent), and *Coelodonta antiquitatis* (woolly rhinoceros, extinct), which together define the Dicerotina clade (supplementary table S1, Supplementary Material online and figs. 3*a* and 4*a*). Nodes 23 and 175 have strictly similar distributions in the studied taxonomic sample even though the former feature is located in the cranium, whereas the latter is in the wrist. Thus, ComponentGrapher allows us to reveal univocal relationships a posteriori between features that were not considered as related in any matter a priori. Such complexes may be synapomorphies of clades, but this is not a necessary condition.

By contrast, disjoint traits are never present in the same organisms, such as the separated metacone and hypocone on the fourth upper premolar (present in the fossil rhinocerotoids *Hyrachyus eximius*, *Trigonias osborni*, *Huaqingtherium lintungense*, and *Aceratherium incisivum*), and the lingual bridge of the protocone and hypocone on the third and fourth upper premolar (present in the recent *Ceratotherium simum* and the extinct *Diceratherium armatum*, *Teleoceras fossiger*, and *Lartetotherium sansaniense*), even though these are two aspects of upper premolar molarization (Antoine 2002) that could be intuitively considered intuitively as evolving interdependently. Indeed, these combinations are not logically incompatible, and they might be found in other rhinocerotoids which were not included in the current taxonomic sample. The protocone and hypocone are lingual cusps, being located antero-lingually and postero-lingually on the tooth, respectively. When they are connected by a thin crest, they form a lingual bridge that is antero-posteriorly oriented. In contrast, the hypocone is the postero-labial cusp. It is either connected to the metacone by a transverse (i.e., labio-lingual) crest, termed metaloph, or not. The lingual bridge and the metaloph are thus not homologous structures. As a consequence, a rhinocerotoid may have at the same time a hypocone separated from the metacone (=metaloph absent) and a lingual bridge on upper premolars; or a metaloph joining a metacone and the hypocone but no lingual bridge (protocone and hypocone are disconnected; the lingual valley is open lingually). Accordingly, these traits may be encoded by genes undergoing antagonistic regulations, or that appeared separately during evolution.

Nested traits, such as the very convex base of the corpus mandibulae present in the closely related rhinocerotids *Ceratotherium simum*, *Diceros bicornis*, and *Coelodonta antiquitatis*, and the rugose frontal bone present in the former taxa, plus their kin *Dicerorhinus sumatrensis* (related to the emblematic diagnostic presence of a frontal horn), provide information regarding the relative stability of traits (fig. 3*b*). Likewise, figure 4*a* illustrates that the complex composed of nodes 23 and 175 is involved in multiple type II relationships. On the one hand, this complex is more broadly distributed than 12 other traits (mostly cranial and mandibular features), such as node 47 (Ramus|‘inclined backward and upward’), which is only found in the “Diceroti” clade. On the other hand, the complex is less widely distributed than 33 other traits (gathering cranial, mandibular, dental, and postcranial [forelimb + hind limb] features). For example, the polyphyletic cluster encompassing Diceroti, the elasmotheriines *Hispanotherium beonense* and *Menoceras arikarense*, and the archetypical teleoceratine *Teleoceras fossiger* contains node 191 (Femur: trochanter major|‘low’) in addition to the complex (fig. 4*b*). This asymmetric taxonomic distribution means that some traits are only present only when another particular trait is also present. Thus, we say that the latter, that is, traits with larger in-degree (number of incoming type II edges), are more stable relative to other traits with which they coexist. Such relatively stable traits are remarkable because they provide a structural backbone, around which the rest of the organismal trait changes. The detection of backbone traits suggests that past organization constraints, and in effect biases, the future evolution of the traits that evolve in organisms. This is understandable from a systemic perspective, that is, central or essential traits, for example, those interacting with many others, have less flexibility to change than traits that are more peripheral in biological organizations. Nested traits can correspond to nested synapomorphies of clades, but this is not always the case.

**Fig. 5.**
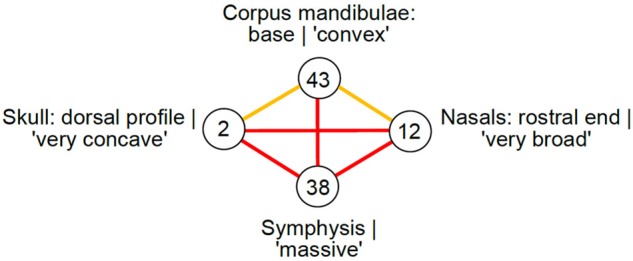
—Example of nodes involved in type III and IV relationships. Node 12: Nasal bones: rostral end|‘very broad’; Node 38: Symphysis|‘massive’; Node 2: Skull: dorsal profile|‘very concave’; node 43: Corpus mandibulae: base|‘convex’. Orange edges correspond to type IV edges, red edges to type III edges. Node 38 is involved in a triangle of type III edges, and occupies what we defined as a central position in 2 type D triplets.

Finally, overlapping traits are distributed across nonnested sets of taxa. For example, the short M1-2 metastyle (discrete dental feature) and the low zygomatic width (with respect to frontal width; metric cranial feature) only occur together in the hyrachyid *Hyrachyus eximius* and the early rhinocerotids *Trigonias osborni* and *Diceratherium armatum*, whereas their evolution is dissociated in other organisms, in which these traits do not co-occur together. Such a distribution is a sign of complex evolution of the traits: it may involve losses, reversions, convergences, and/or parallelisms. When three traits have a type III relationship, they form a triangle in the type III trait network. A triangle means that the evolution of these traits is dissociated in at least some taxa, and suggests that the presence of these traits is not under a common developmental regulation over evolutionary time. Thus, nodes 2 (Skull: dorsal profile|‘very concave’), 38 (Symphysis|‘massive’), and 12 (Nasal bones: rostral end|‘very broad’) display such a complicated distribution, indicating that rhinos can contain a mosaic of these traits (fig. 5), which appear developmentally dissociable from one another. A high proportion of triangles in the type III trait network thus means that a high proportion of traits can evolve in such a dissociated fashion. Therefore, it provides a measure of general dissociability. We refer to organismal fluidity as the case when the same traits (rather than different traits) are found in distinct combinations. Organismal fluidity is higher when the proportion of triangles is higher, that is, when the type III networks increasingly resemble a clique because the highest proportion of triangles occurs when all nodes are connected together by a type III edge in the graph. This fluidity should not be confused with the dissociations of genes produced by introgressive processes in prokaryotic taxa. Unless hybridization or introgression occurred (Mallet et al. 2016), the multiple traits of a given “fluid” metazoan are likely derived from a single common ancestor. However, the genes encoding for these traits, and thus the interactions between them, have not necessarily been subjected to simultaneous regulation, activation, and inactivation during organismal evolution, which decouples their presence in different organismal lineages.

Finally, some traits (central in type D triplets, fig. 2) are alternatively found with traits that never occur together. For example, the trait *t* appears pivotal, whereas the traits *s* and *u* appear potentially excludable. Thus, pivotal traits may be involved in distinct morphological organizations. This behavior is an extreme form of versatility. Pivotal traits may typically have been co-opted for novel functions, or may have contributed to a switch (Tanay et al. 2005) in organization (e.g., the interaction between *s* and *t* may have switched to an interaction between *t* and *u*). The morphological organizations including a given pivotal trait are more different when there are more type D triplets centered on that pivotal trait. The detection of pivotal traits is a precondition to evaluate their role during organismal evolution. For example, figure 5 shows how node 38 (Symphysis|‘massive’), broadly distributed in clade including almost all Rhinocerotinae (*Dicerorhinus sumatrensis*, *Teleoceras fossiger*, *Diaceratherium aginense*, *Lartetotherium sansaniense*, *Hoploaceratherium tetradactylum*, *Aceratherium incisivum*, *Diaceratherium lemanense*, *Rhinoceros unicornis*, *Rhinoceros sondaicus*) is involved in 2 type D triplets. First, node 38 is connected to node 43 (Corpus mandibulae: base|‘convex’) in early Elasmotheriines (*Hispanotherium beonense*, *Huaqingtherium lintungense*, *Menoceras arikarense*) plus *Teleoceras fossiger* clade and to node 2 (Skull: dorsal profile|‘very concave’) in living African rhinos clade (*Ceratotherium simum*, *Diceros bicornis*, *Lartetotherium sansaniense*, *Rhinoceros unicornis*). Second, node 38 is connected to node 43 (Corpus mandibulae: base|‘convex’) in early Elasmotheriines plus *Teleoceras fossiger* clade and to node 12 (Nasal bones: rostral end|‘very broad’) in living African rhinos clade. Thus, a massive symphysis can be used as a backbone for different morphological organizations, but these organizations cannot simultaneously contain: 1) a very dorsal concave profile and a jaw with a convex base, nor simultaneously present, or 2) a jaw with a convex base and nasals with a very broad dorsal end.

## Results

### Rhinocerotid Evolution from a Network Perspective

We considered and recoded the data set primarily modified from (Antoine 2002; Antoine et al. 2003), describing 120 traits present in 21 taxa of ceratomorph mammals. There were no missing data, with the exception of one character state in the taxa *Huaqingtherium lintungense*. These characters are primarily focused on rhinocerotids (rhinos) within ceratomorph perissodactyls. This data set includes 15 fossil species and six members of extant lineages among rhinos and tapirs (see Materials and Methods and supplementary data set S1, Supplementary Material online). We detected eight complexes, which does not differ from expectations by chance according to both null models (table 1). Finding complexes opens the intriguing possibility that maybe some character states that appeared to belong to different characters are in fact inseparable instances of a common developmental regulatory pathway. They may include a single character that was not previously characterized as such, in particular for complexes present in multiple organisms. It is of course for the experts to determine whether they want to use the detection of unexpected complexes in this way, particularly for the six complexes, which associated traits from different regions of the body plan, such as cluster 6: presence of the third inferior incisor + presence of an inferior canine + astragalus higher than wide (fig. 3*a*). If the two first dental features (coinciding with adjacent loci on the same bone) may not be fully independent, there is no reason a priori to consider that they would be associated with changes in the proportion of the central ankle bone on the hind limb.

**Table 1:** Summary of Network Metrics with Results of Corresponding Permutation Test for Rhinocerotoids

Trait Networks	Network Metrics	Reference Value	Equiprobable Model	Significance	Phylogenetics Model	Significance
Type I network	Number of complexes	8	0.040191962	NS	0.041693953	NS
	Number of edges	22	0.00079984	NS	0.000218293	Higher
Type II network	Number of directed edges	5,100	0.00019996	Higher	0.000218293	Higher
	Number of significantly stable traits	50				
Type III network	Number of edges	16,063	0.00019996	Lower	0.000218293	Lower
	Number of triangles	680,642	0.00019996	Lower	0.000218293	Lower
	Proportion of triangle	0.4286196	0.00019996	Lower	0.000218293	Lower
	Density	0.711444795	0.00019996	Lower	0.000218293	Lower
Type IV network	Number of edges	4,774	0.00019996	Higher	0.000218293	Higher
Type II+IV network	Number of type D triplets	186,504	0.00179964	NS	0.000873172	NS
	Number of significantly pivotal traits	21				

Note.—*P* values were adjusted for multiple tests with a Bonferroni correction.

Higher, significantly higher than expected by chance; lower, indicates significantly lower than expected by chance; NS, nonsignificant.

More precisely, complexes occur at both terminal (1, 3, 4, 6, and 8) and internal nodes (2, 5, and 7). They are mainly documented in the subfamily of living rhinos, the Rhinocerotinae. Within the latter clade, Miocene Aceratheriini (extinct hornless rhinos) has two dental-based complexes (complexes 7 and 8) and the short-limbed and hippo-like teleoceratine *Brachypotherium brachypus* yields a jaw- and teeth-based complex (complex 4). Two-horned rhinos, either living (Sumatran, white and black rhinos) or recently extinct (woolly rhino), comprise more integrative complexes, containing skull and tooth characters (complexes 1 and 3), skull and forelimb characters (complex 2). The most inclusive complex (complex 5) encompasses jaw, tooth, and forelimb features, observed in the morphologically well-supported woolly, white, and black rhino clade (Antoine 2002). Interestingly, the woolly rhino is not closely related to the African rhino clade, but sister taxon to the Sumatran rhino instead, in most molecular phylogenies (Kosintsev et al. 2019). Conversely, no complex characterizes the early diverging sister group to Rhinocerotinae, that is, Elasmotheriinae. At first sight, all complexes located at internal nodes involve closely related taxa: that is, complexes 2 (two-horned rhinos), 5 (grazers among two-horned rhinos), and 7 (Aceratheriini). In other words, they may be good indicators of strongly supported morphological clusters. Moreover, one complex concerns the nonrhinocerotid taxa of the rhino data set, that is, the outgroups (the extant Brazilian tapir *Tapirus terrestris* and the early diverging hyrachyid *Hyrachyus eximius*) gathering tooth and hind limb characters.

All of these complexes are small, associating at most four traits. Collectively, complexes encompass a total of 22 traits, that is, <18% of all traits. Thus, they represent only limited portions of these organisms. Therefore, most described traits of rhinos happen to be dissociated during evolution. Consistently, there are 5,100 type II edges, which is significantly higher than expected by chance according to both null models. We tested whether these nested distributions of traits correspond to synapomorphies along the tree of rhinos. Only eight (0.16%) of the pairs of traits with nested distributions are hosted by nested clades, whereas 492 (9.6%) are hosted by a clade included in a paraphyletic group. Moreover, 4,600 (90%) of the pairs of traits with nested distributions correspond to two nested paraphyletic groups. Thus, nested traits of rhinos cannot usually be simply explained by the evolution of synapomorphies and as such their remarkable associations must be accounted for by additional processes. For example, a distal articulation strongly oblique with respect to the trochlea on the astragalus (ankle bone) convergently evolved with the orientation of lower molar hypolophids, yet the latter never existed without the former (fig. 3*b*). Interestingly, there was no reason to consider these postcranial and dental features as being related a priori.

Detailed analysis of type II edges, contrasting in-degrees and out-degrees for all traits of the network, shows that the organization of traits forming rhinocerotoids is rather labile. Fifty traits however were significantly more stable relatively to other traits than expected by chance according to both null models (supplementary table S2, Supplementary Material online), constituting a detectable backbone in rhinos. The majority of these significantly stable traits involves character states from different characters, indicating that a minority of the characters of rhinos are structurally more stable. Among them, there is a predominance of “iconic” features (e.g., nasal and frontal horns, crown height, dental formula, shape of the last upper molar, and tridactyl hand), considered as diagnostic in pre-Hennigian/phylogenetic classifications, whereas phylogenetic analyses based on equivalent data sets have demonstrated that these traits are strongly influenced by convergence and/or parallelism (Antoine 2002; Antoine et al. 2003, 2010; Becker et al. 2013). In other words, these traits seem to be relevant for understanding the rhinocerotoid body plan, even though they appear less useful for classic phylogenetic analyses. Typically, 18 of these traits were couplets, such as the narrow and the very broad rostral ends of the nasal bones, reflecting the stability of a minority of structurally coupled characters.

**Fig. 6.**
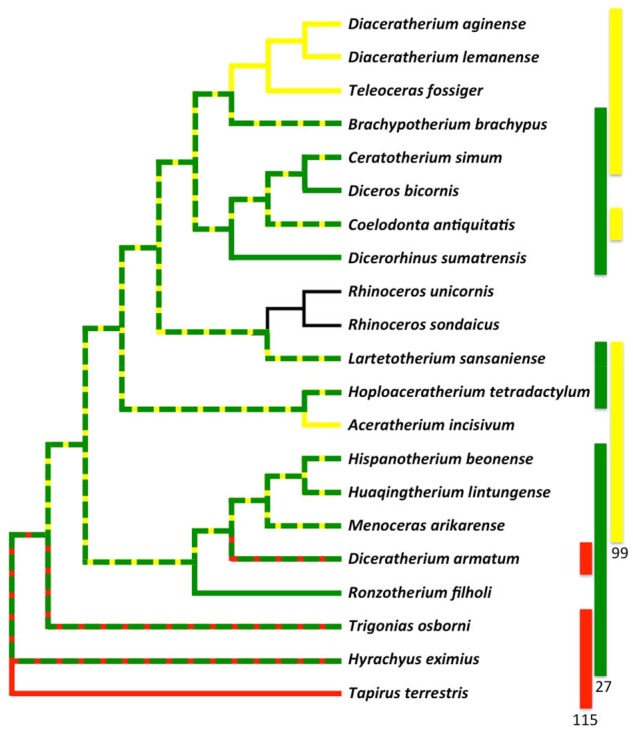
—Mapping of a type D triplet along the phylogeny of rhinocerotids. Each trait is represented by a different color. The distribution of trait 27 overlaps with that of trait 99; the distribution of trait 27 overlaps with that of trait 115; however, the distributions of trait 99 and 115 are disjoint. 27: Zygomatic/frontal widths|‘less than 1.5’; 99: Upper molars: crochet|‘always present’; 115: M1-2: metastyle|‘short’.

Additionally, there were 16,063 type III edges in the trait network. Although significantly less abundant than expected by chance, these relationships provide supplemental evidence of the general dissociability of traits during rhinocerotoid evolution. For example, a low zygomatic width (with respect to frontal width) is sometimes (but not always) associated with a short metastyle on the first-second upper molars, and sometimes (but not always) it is associated with a crochet on upper molars. However, no rhinos harbor both a crochet on upper molars and a short metastyle, suggesting that a low zygomatic width can be a pivotal feature between different morphological organizations (fig. 6). Consistently, other metrics of the trait network show that the evolution of rhinos frequently involved similar traits albeit in different combinations in different organisms. For example, focusing on type III edges, the density of type III edges reaches 0.71, the proportion of triangles constituted by type III edges reaches 0.43, and the diameter, defined as the longest of the shortest paths between any pair of traits, is 2. Interestingly, 21 traits, such as the foramen mentale in front of p2 or at the level of p2-4, are pivotal and appear significantly overrepresented at the center of type D triplets (supplementary table S3, Supplementary Material online). These pivotal traits were in large majority couplets (16 out of 21; mainly on teeth, and to a lesser extent on jaw and limbs; e.g., tibia and fibula independent or fused). Be they plesiomorphic or derived states (Antoine 2002), these features have taken part in distinct morphological organizations among rhinocerotoids.

**Fig. 7.**
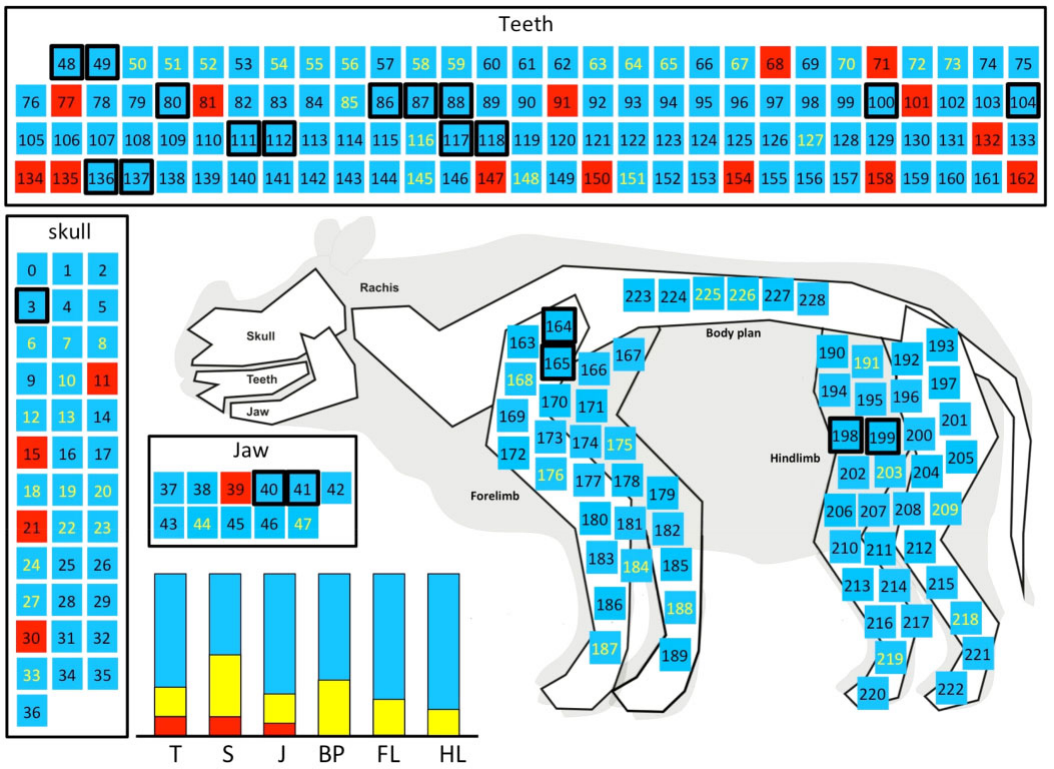
—Schematic mapping of morphological traits on the rhinocerotoid body plan. Main regions are indicated in boxes. Red squares are relatively unstable traits (i.e., type II in-degree is null); blue squares are relatively stable traits (i.e., type II in-degree is positive); yellow squares indicate traits with significant relative stability (*P* value <0.05, following a Bonferroni correction for multiple tests, equiprobable and phylogenetic permutation tests). Numbers in squares correspond to NodeID. Black boxed squares correspond to traits that are significantly central in type D triplets (*P* value <0.05, following a Bonferroni correction for multiple tests, equiprobable and phylogenetic permutation tests). The barplot indicates the relative frequencies of traits in main regions of the rhinocerotoid body plan, observed in all species. Areas in red/blue/yellow are versatile/relatively stable/significantly stable traits, respectively. The main regions are T, teeth; S, skull; J, jaw; BP, body plan; FL, forelimb; and HL, hind limb.

Overall, mapping unstable, stable, significantly stable and pivotal traits into the body plans of rhinocerotoids allowed us to analyze whether in different regions of the body plan the morphology is affected by different evolutionary processes. Mapping the traits on the rhino body plan demonstrated regionalization of unstable traits (i.e., relatively to other traits) (Fisher exact test, *P* value 0.05) (fig. 7). These unstable traits were significantly more abundant in the cranio-dental region (ca. 10% of cranio-mandibular and dental features) than in the postcranial region. Interestingly, unstable traits consist of independent characteristics or singletons, instead of couplets. The total absence of unstable characters recognized for the body plan or the limb bones (0/66) was striking. The postcranial skeleton is indeed remarkably stable within the controlled rhinocerotoids with respect to the cranio-mandibular region and teeth, pointing to an early implementation of the postcranial Bauplan among rhinocerotoids, without major changes since then. This contrasts with the results regarding the distribution of homoplasy in phylogenetic analyses focused on similar data sets (Antoine 2002; Antoine et al. 2010; Becker et al. 2013), where all the considered body regions yield a similar amount of homoplastic characters. Therefore, trait network analysis shows that morphological instability does not equal homoplasy and that the network- and phylogenetic-based approaches are complementary in depicting distinct aspects of trait versatility.

## Discussion

Our approach provides a new strategy for complementary reanalyses of currently available data from a systemic perspective, in particular palaeontological data. Of note, alternative approach would have certainly been possible to analyze traits distributions across taxa using networks. Here, we chose to use an intuitive method that takes advantage of a multiplex graph, which only requires the definition of four types of relationships between the distributions of pairs of traits (nested-identical-overlapping-disjoint). We selected this approach based on the comparison of splits of traits, firstly, because we thought it would be very natural to phylogeneticists traditionally working with trees. For example, in the context of bootstrap analyses, phylogeneticists are familiar with the need to compare splits of taxa, that is, to identify identical splits (akin to our type I edges), and compatible and nested splits (akin to our type II edges) from a list of splits generated from different bootstrapped trees. Indeed, these two kinds of splits of taxa are typically the ones that enhance the support for a phylogeny. Likewise, phylogeneticists who have used splitnetworks, as a way to explicitly represent the presence of compatible and incompatible splits of taxa would intuitively appreciate the use of multiple types of edges. Users of single layer splitnetworks understand that overlapping splits of taxa (akin to our type III edges) will produce a reticulate pattern in their graph. Finally, beyond the simplicity of defining multiple types of edges to analyze traits distributions, we relied upon multiplex graphs, because this formalism allowed us to perform analyses of colored motifs (such as the search for type D triplets), as well as some specific analyses; for example, in-degree/out-degree analyses for nodes connected by type II edges. This would not have been possible in a single layer network, for example, in a typical co-occurrence networks, in which a single correlation statistic could result from distinct patterns of distributions of traits between pairs of taxa, or in a splitnetwork in which such motifs are by definition absent.

Many network tools and libraries could be used to analyze these traits networks (such as networkX or possibly Gephi or Cytoscape to compute transitivity/average clustering coefficient for type III graphs). The added value of ComponentGrapher is that it does not start with these trait networks, but it builds them from a classic data matrix. Then, in a single analysis, ComponentGrapher computes all relevant indices on all types of networks. It handles type I, type II, type III, and type IV networks, separately, as well as the multiplex network, even though some of these graphs (type I, III, and IV) are undirected networks, while the others (the multiplex and type II networks) are directed. Moreover, ComponentGrapher searches for all relevant motifs in these diverse networks, in particular type D triplets, which are not implemented elsewhere. Furthermore, ComponentGrapher implements two kinds of statistical tests of the significance values of these various network measures, including a phylogenetically informed test, which no other software/network tools produces. iv) Finally, ComponentGrapher organizes these topological indices into structured outfiles, so that any biologist owning a nexus or phylip format file can run a full trait network analysis even without being a skilled programmer, whereas a skilled programmer can even go one step further and exploit ComponentGrapher outfiles, for example, to compare the taxa distribution associated with traits associations with a reference phylogeny, when such a phylogeny is known.

Our network analyses describe how associations of evolved traits can contribute to a mechanistic explanation of evolution. These results confirm that not all components of the anatomy of a given organism change at the same time, at the same rate, or in the same way, but likely as a result of various structural constraints, and that this heterogeneity of modes of evolution can probably not be captured by evolutionary models that treat characters as if they were evolving independently, because the uncoordinated model of trait evolution was rejected. Moreover, our method highlighted traits with remarkable behavior during evolution, in terms of their relative stability, their pivotal distribution, and their contribution to complexes. Relatively less stable traits are only observed in the heads of rhinos. Moreover, the general observation that many of these animal traits are used repeatedly, in different combinations, in different taxa, which usually do not form clades, suggests that the genes encoding these traits might be inherited without expression (or lost by genetic drift) from a common ancestor, and might be recruited into novel gene regulation networks during the course of evolution. Alternative explanations would be that similar morphological traits can be invented on multiple occasions and coded from different gene sets, or that traits losses are massive during organismal evolution.

The former interpretations agree with the description of the main developmental stages in terms of gene regulatory networks proposed in the pioneering work of Britten and Davidson (Britten and Davidson 1969), now theoretically and experimentally validated (Gao and Davidson 2008; Davidson 2010; Peter and Davidson 2011; Erkenbrack and Davidson 2015; Gillis and Hall 2016). As stated by (Davidson 2010), “*it is obvious that if there is indeed a finite repertoire of network sub-circuits used to effect development, the evolution of development has to be considered as the process of assembly, reassembly, and redeployment of these sub-circuits.*” Aspect of morphology should reflect this genomic fluidity. Therefore, analyses of palaeontological data with trait networks could allow generation of hypotheses about the role of important aspects of developmental evolution, namely regulation and heterochrony, in evolutionary changes, when the resulting network patterns suggest frequent parallelism and convergence. Consequently, our analysis encourages an openly pluralistic modeling of organismal evolution, including trees and networks, and supports the coupling of constitutes developmental and palaeontological studies. Likewise, in trait network analyses, behavioral traits could be included together with morphological traits to capture relationships spanning over the whole organismal phenotype. Such an approach does not diminish the importance of phylogenetic reconstruction, but rather stresses the need for further integration of network-thinking into evolutionary analyses (Wilkins 2007), because it has the potential to enhance the retrodictive dimension of evolutionary biology. Moreover, phylogenetic inferences could enhance the construction of trait networks. For example, the distribution of inferred ancestral character states along a species tree (using BEAST, Bouckaert et al. 2014; or MrBAYES, Ronquist and Huelsenbeck 2003, for example) could provide valuable input data for a trait network analysis. In this case the trait network could be seen as a posttreatment of the phylogenetic-based inference and used to represent what character states were found together, decoupled or disjoint according to BEAST or MrBAYES analyses. The topology of such trait networks could then be investigated.

Because our graph-theoretical approach investigates types of trait distribution (or more generally components) in higher level structures, without the need for an underlying phylogeny, it could be used to analyze organizations from the molecular level (i.e., by analyzing the distributions of proteins across organellar proteomes) up to the ecosystemic level (i.e., by analyzing the distributions of OTUs or species across environmental samples). In this type of “-omics,” the types (and amount) of data to be compared between taxa are increasing at a rate that is faster than the implementation of accurate evolutionary models to describe their behavior. In that sense, networks (be they multiplex or single layer graphs) can contribute to further integration of systems and evolutionary biology. We believe such an evo-systemic could be particularly informative because evolution from molecules to ecosystems depends on the changes in organization as well as on the divergence and merging of lineages. 

## Supplementary Material

Supplementary data are available at *Genome Biology and Evolution* online.

## Supplementary Material

evz182_Supplementary_Data
