# Supplementary material for: Introducing Trait Networks to Elucidate the Fluidity of Organismal Evolution Using Palaeontological Data
Source: Genome Biol Evol. 2019 Sep 5;11(9):2653–65. doi: 10.1093/gbe/evz182 (PMC6761957; doi:10.1093/gbe/evz182)
Supplement: evz182_Supplementary_Data [file evz182_supplementary_data.pdf]

Algorithm 1: Algorithm to compute the co-occurrence network from a character matrix

```
function ComputeNetwork (M)
  Input  : matrix M(T,C) of T taxa (rows) and C characters (columns)
  Output: a co-occurrence network N(n,e) with n nodes and e edges
Compute each node n and associated encoding
for i ← 1 to len(C) do
    foreach unique valid¹ character s of the column C[i] do
        new node n= Encode(C[i],s)
        n.column = i
        Add node n to network N
    end
end
Compute each edge e
foreach node n1 associated with column i do
    foreach node n2 associated with column j do
        /* edges are undirected unless otherwise stated */
        new edge e
        X=n1.states ∩ n2.states
        if X = ø then
            e.type = 4 (disjoint)
        else if n1.total=n2.total=|X| then
            e.type = 1 (identical)
        else if |X| = n1.total and |X| < n2.total then
            /* directed edge from n1 → n2 */
            e.type = 2 (Inclusion)
        else if |X| = n2.total and |X| < n1.total then
            /* directed edge from n2 → n1 */
            e.type = 2 (Inclusion)
        else
            e.type = 3 (Overlap)
        end
        Add edge e to network N
    end
end
return network N
¹Valid character are {0..9, A..Z}. Unvalid characters {$, * and -} are
 ignored during the node creation while polymorphic character e.g. {0,1}
 at a unique position in matrix M must either be selected or removed
 prior to running the algorithm.
```

Algorithm 2: Algorithm to create a new node n with associated binary states

```
function Encode (c,s)
  Input  : column c of matrix M, character s
  Output: new node
new node n
n.states ← {}
n.total = 0 /* total taxa associated with this node */
for i ← 1 to len(c) do
    if c[i] = s then
        n.states[i] =1
        n.total++
    else
        n.states[i] =0
    end
end
return node n
```

Fig. S1. Pseudocode of the two algorithms used for this analysis

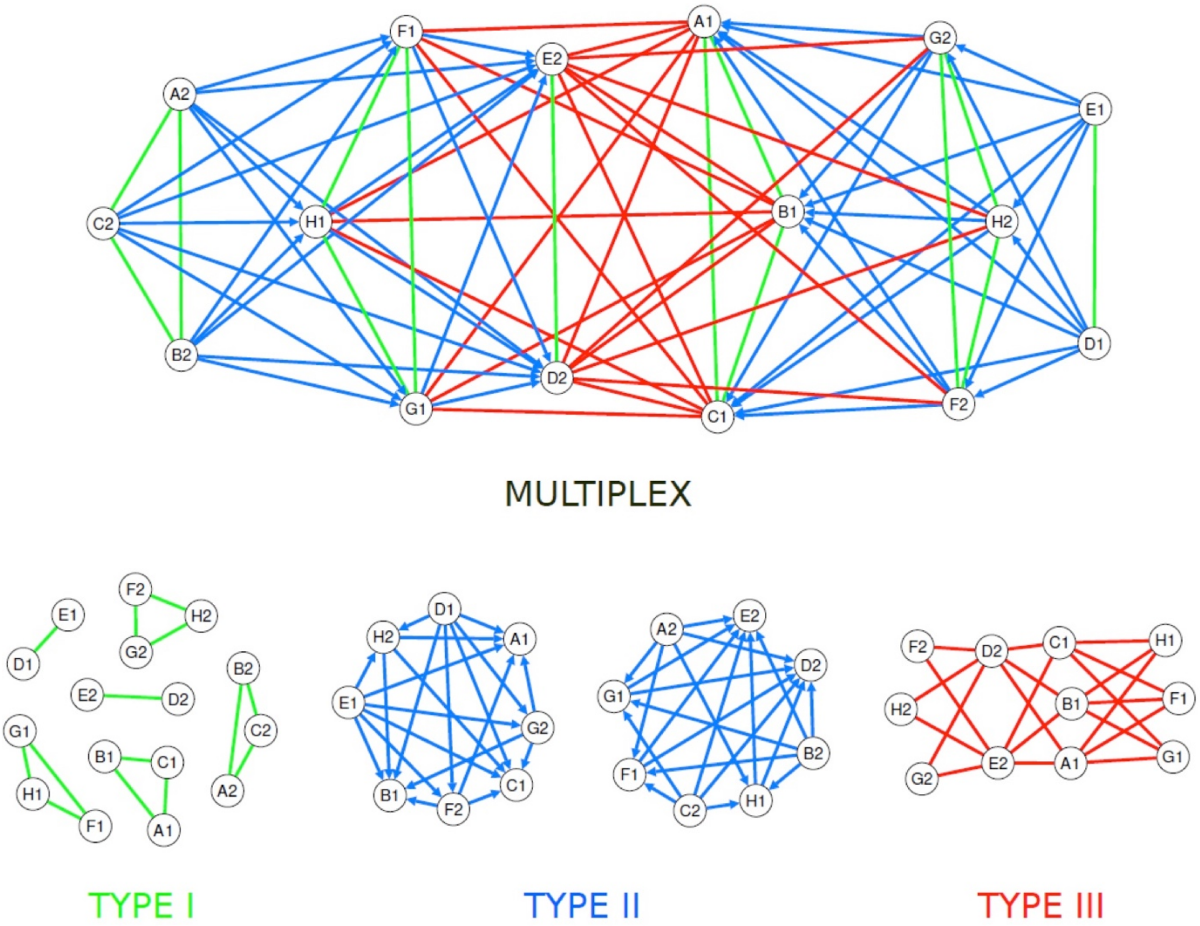

Fig. S2. Co-occurrence networks for paleontological studies. Each trait is treated as an individual node. Two nodes are directly connected by an edge indicating their type of relationship (I, II or III), but are disconnected otherwise (Type IV). This inclusive co-occurrence graph can also be decomposed into three networks: a green network of identity featuring only nodes connected by type I edges; a blue network of inclusion featuring only nodes connected by oriented type II edges, an arrow pointing from the least stable toward the most stable trait; a red network of overlaps featuring only nodes connected by type III edges.

Table S1. Relationships between connected traits in type I graphs. This table describes the composition and distribution of the complexes detected in our analysis.

<table>
<tr><th>Complexes</th><th>Node ID</th><th>Character</th><th>State</th><th>Description | State</th><th>Taxa</th></tr>
<tr><td>1</td><td>15</td><td>6</td><td>2</td><td>Nasal bones| 'fused '</td><td>Coelodonta antiquitatis</td></tr>
<tr><td>1</td><td>77</td><td>37</td><td>3</td><td>P3-4: crochet and crista| 'always joined'</td><td>Coelodonta antiquitatis</td></tr>
<tr><td>1</td><td>101</td><td>48</td><td>3</td><td>Upper molars: crista| 'always present '</td><td>Coelodonta antiquitatis</td></tr>
<tr><td>1</td><td>132</td><td>62</td><td>1</td><td>M3: protocone| 'usually unconstricted'</td><td>Coelodonta antiquitatis</td></tr>
<tr><td>2</td><td>23</td><td>10</td><td>1</td><td>Frontal bone: aspect | 'rugose '</td><td>Ceratotherium simum,Diceros bicornis,Dicerorhinus sumatrensis,Coelodonta antiquitatis</td></tr>
<tr><td>2</td><td>175</td><td>85</td><td>2</td><td>Pyramidal: distal facet for semilunate | 'L-shaped '</td><td>Ceratotherium simum,Diceros bicornis,Dicerorhinus sumatrensis,Coelodonta antiquitatis</td></tr>
<tr><td>3</td><td>30</td><td>13</td><td>2</td><td>Occipital crest | 'forked '</td><td>Ceratotherium simum</td></tr>
<tr><td>3</td><td>134</td><td>63</td><td>1</td><td>M3: protoloph | 'mesiodistally elongated '</td><td>Ceratotherium simum</td></tr>
<tr><td>3</td><td>162</td><td>78</td><td>2</td><td>Lower molars: hypolophid | 'almost mesiodistally oriented '</td><td>Ceratotherium simum</td></tr>
<tr><td>4</td><td>39</td><td>17</td><td>2</td><td>Symphysis | 'very massive '</td><td>Brachypotherium brachypus</td></tr>
<tr><td>4</td><td>154</td><td>74</td><td>1</td><td>p2: paraconid | 'reduced '</td><td>Brachypotherium brachypus</td></tr>
<tr><td>5</td><td>44</td><td>19</td><td>2</td><td>Corpus mandibulae: base | 'very convex '</td><td>Ceratotherium simum,Diceros bicornis,Coelodonta antiquitatis</td></tr>
<tr><td>5</td><td>47</td><td>20</td><td>2</td><td>Ramus| 'inclined backward and upward'</td><td>Ceratotherium simum,Diceros bicornis,Coelodonta antiquitatis</td></tr>
<tr><td>5</td><td>109</td><td>51</td><td>1</td><td>M1-2: protocone| 'usually unconstricted'</td><td>Ceratotherium simum,Diceros bicornis,Coelodonta antiquitatis</td></tr>
<tr><td>5</td><td>177</td><td>86</td><td>1</td><td>Pyramidal: distal side | 'elliptic '</td><td>Ceratotherium simum,Diceros bicornis,Coelodonta antiquitatis</td></tr>
<tr><td>6</td><td>60</td><td>28</td><td>0</td><td>i3 | 'present '</td><td>Tapirus terrestris,Hyrachyus eximius</td></tr>
<tr><td>6</td><td>61</td><td>29</td><td>0</td><td>c | 'present '</td><td>Tapirus terrestris,Hyrachyus eximius</td></tr>
<tr><td>6</td><td>204</td><td>101</td><td>0</td><td>Astragalus: TD/H | 'TD/H<1 '</td><td>Tapirus terrestris,Hyrachyus eximius</td></tr>
<tr><td>7</td><td>66</td><td>32</td><td>1</td><td>P2-4: metaloph |constricted</td><td>Hoploaceratherium tetradactylum,Aceratherium incisivum</td></tr>
<tr><td>7</td><td>106</td><td>50</td><td>1</td><td>Upper molars: lingual cingulum | 'usually present '</td><td>Hoploaceratherium tetradactylum,Aceratherium incisivum</td></tr>
<tr><td>8</td><td>91</td><td>43</td><td>2</td><td>P4: antecrochet | 'usually present '</td><td>Hoploaceratherium tetradactylum</td></tr>
<tr><td>8</td><td>135</td><td>64</td><td>1</td><td>p2-3: vertical external rugosity | 'present '</td><td>Hoploaceratherium tetradactylum</td></tr>
</table>

Table S2. Nodes with significantly high in-degree in type II graphs. This table describes the traits with significantly higher in-degree in the type II graph than in random networks constructed using two different null permutation models.

NodeID	Character-state	Indegree (type II network)
6	Skull: back of tooth row|'in the posterior half'	104
7	Skull: back of tooth row|'in the anterior half'	4
8	Skull|'dolichocephalic'	16
10	Nasal bones: rostral end|'narrow'	40
12	Nasal bones: rostral end|' very broad'	12
13	Nasal bones|'totally separated'	35
18	Nasal bones (median region): aspect|smooth	35
19	Nasal bones (median region): aspect|rugose	24
20	Nasal bones (lateral edges): aspect|straight	103
22	Frontal bone: aspect|smooth	71
23	Frontal bone: aspect|'rugose'	12
24	Orbit|'not laterally projected'	82
27	Zygomatic/frontal widths|'less than 1.5'	35
33	Squamosal: processus zygomaticus|flat	45
44	Corpus mandibulae: base|'very convex'	8
47	Ramus|'inclined backward and upward'	8
50	Cheek teeth|'without cement'	23
51	Cheek teeth|'with cement'	27
52	Cheek teeth: aspect of the enamel|'wrinkled'	15
54	Cheek teeth: aspect of the enamel|'corrugated and arborescent'	14
55	Cheek teeth: crown|'low'	37
56	Cheek teeth: crown|'high'	19
58	i1|'present'	62
59	i2|'present'	68
63	Upper premolars: labial cingulum|'usually present'	5
64	P2-4: crochet|'usually present'	45
65	P2-4: metaloph|unconstricted	108
67	P2-4: lingual cingulum|'always present'	31
70	P2-4: postfossette|'wide'	11
72	P1 (in adults)|'always persistent'	27
73	P1 (in adults)|'usually persistent'	13
85	P3-4: metaloph|'hypocone posterior to metacone'	54
116	M1-2: metastyle|'long'	82
127	M3: ectoloph and metaloph|'fused (ectometaloph)'	84
145	Lower premolars: lingual opening of the posterior valley (lingual view)|'V-shaped'	35
148	Lower premolars: labial cingulum|'present'	20
151	p2: paralophid|'curved	28
168	Semilunate: ulna-facet|'present'	21
175	Pyramidal: distal facet for semilunate|'L-shaped'	12
176	Pyramidal: distal side|'triangular'	94
184	McIII: magnum-facet in anterior view|'visible'	71
187	McV|'vestigial'	45
188	Metacarpals: insertion of the m. extensor carpalis|'flat'	36
191	Femur: trochanter major|'low'	19
203	Tibia: posterior apophysis|'rounded'	44
209	Astragalus: orientation of the fibula-facet|'oblique'	29
218	Astragalus: orientation trochlea/distal articulation|'same axis'	39
219	Astragalus: calcaneus-facet 1|'very concave'	89
225	Metapodials: intermediate reliefs|'high and acute'	40
226	Metapodials: intermediate reliefs|'low and smooth'	20

Table S3. Node with significant centrality in type D triplets. This table describes the traits with significant centrality in the type III+IV graph than in random networks constructed using two different null permutation models.

NodeID	Character-state	Number of times central
3	External auditory pseudo-meatus|'open '	1930
40	Foramen mentale|'in front of p2 '	2173
41	Foramen mentale|'at level of p2-4 '	2173
48	Compared length of P-p/M-m|'(100 * LP3-4/LM1-3)>50'	1880
49	Compared length of P-p/M-m|'42<(100 * LP3-4/LM1-3)<50 '	1880
80	P3-4: protocone and hypocone|'separated '	1879
86	P3: protoloph|'joined to the ectoloph '	448
87	P3: protoloph|'interrupted '	448
88	P3: crista|'usually present '	1374
100	Upper molars: crista|'usually present '	1223
104	Upper molars: crochet and crista|'usually separate'	508
111	M1-2: paracone|folded	452
112	M1-2: paracone|unfolded	452
117	M1-2: metaloph|'long '	1869
118	M1-2: metaloph|'short '	1869
136	Lower cheek teeth: trigonid|'rounded '	1841
137	Lower cheek teeth: trigonid|'sharp-edged'	1841
164	Scaphoid: magnum-facet in lateral view|'straight '	1077
165	Scaphoid: magnum-facet in lateral view|'concave '	1077
198	Tibia-fibula|'independent '	1348
199	Tibia-fibula|'in contact or fused '	1348
